# Mechanical Failure Mode of Metal Nanowires: Global Deformation versus Local Deformation

**DOI:** 10.1038/srep11050

**Published:** 2015-06-18

**Authors:** Duc Tam Ho, Youngtae Im, Soon-Yong Kwon, Youn Young Earmme, Sung Youb Kim

**Affiliations:** 1 Department of Mechanical Engineering, Ulsan National Institute of Science and Technology, Ulsan 689-798, South Korea; 2 Department of Mechanical Engineering, Korea Advanced Institute of Science and Technology, Daejeon 305-701, South Korea; 3 School of Materials Science and Engineering, Ulsan National Institute of Science and Technology, Ulsan 689-798, South Korea; 4 Multiscale and Multiphysics Simulation Group and Low-Dimensional Carbon Material Center, Ulsan National Institute of Science and Technology, Ulsan 689-798, South Korea

## Abstract

It is believed that the failure mode of metal nanowires under tensile loading is the result of the nucleation and propagation of dislocations. Such failure modes can be slip, partial slip or twinning and therefore they are regarded as local deformation. Here we provide numerical and theoretical evidences to show that global deformation is another predominant failure mode of nanowires under tensile loading. At the global deformation mode, nanowires fail with a large contraction along a lateral direction and a large expansion along the other lateral direction. In addition, there is a competition between global and local deformations. Nanowires loaded at low temperature exhibit global failure mode first and then local deformation follows later. We show that the global deformation originates from the intrinsic instability of the nanowires and that temperature is a main parameter that decides the global or local deformation as the failure mode of nanowires.

Mechanical responses of metal nanowires have been extensively studied since they show unusual behaviors such as very large elastic range and higher elastic moduli compared to their bulk counterparts. In particular, failure event of metal nanowires under uniaxial tensile loading has been of great interest, because it gives us fundamental understanding about what makes the nanowires fails, when the nanowire fails, and what deformation the nanowire undergoes in that moment. Therefore, a lot of simulations^1^,^2^,^3^,^4^ and experiments^5^,^6^,^7^,^8^ have been conducted during last two decades, and it has been believed that metal nanowires fail by the occurrence of local deformations such as full slip, partial slip or twinning. During loading, nanowires are under the competition among different local deformations and finally fail by one of them. Final failure deformation of nanowires is dependent on many parameters such as stacking fault energy^9^, orientation of nanowire^10^, twinability^11^, orientation of side facet^12^, and Schmidt factor^3^. In addition, nanowires can fail with ductile necking or simple shear brittle failure depends on the length of nanowires^1^.

Most predominant origin of local deformations that makes nanowires fail is the nucleation and propagation of dislocations. Since the energy barrier for the nucleation of dislocation is lower at edges than on surfaces or in bulk, the dislocation initiates from one of edges of the nanowire, propagates through the cross-section, and finally forms one of local deformations^4^,^13^. However, we provide here numerical and theoretical evidences that local deformation is not the one and only failure mode of nanowires. Rather, a global deformation, that has no relation with dislocations, is another failure mode of metal nanowires. In addition, we find that there is a competition between local and global deformations and that temperature is a primary parameter which decides the failure mode of nanowires. The basic mechanism of the global failure mode can be explained by the elastic instability theory that was originally developed by Born^14^, and then improved by Hill and Milstein^15^,^16^ and others^17^,^18^,^19^.

## Results

### Failure mode of Au [001]/(001) nanowires

In order to find the failure mode of metal nanowires, we performed uniaxial tension tests for metal nanowires by using molecular dynamics (MD) simulation. The face-centered-cubic (FCC) (001) nanowires of seven metals were modelled with embedded atom method (EAM) potentials^20^,^21^,^22^. For convenience of notation, we assigned the *x*-direction to be the length direction (the [100]-direction), and *y*- and *z*-directions to be the lateral directions (the [010]- and [001]-directions, respectively). Periodic boundary condition was applied in the length direction. To study the temperature effect on the tensile tests of nanowires, we assigned the temperature to the nanowires ranged from 0.01 K to 300 K using Nosé-Hoover thermostat^23^,^24^. We first equilibrated the nanowires at a target temperature for 100,000 timesteps and applied strain in the length direction with a strain rate of 10^8^ s^-1^. Timestep was 1 femtosecond and all calculations were conducted by LAMMPS^25^.

**Figure 1:**
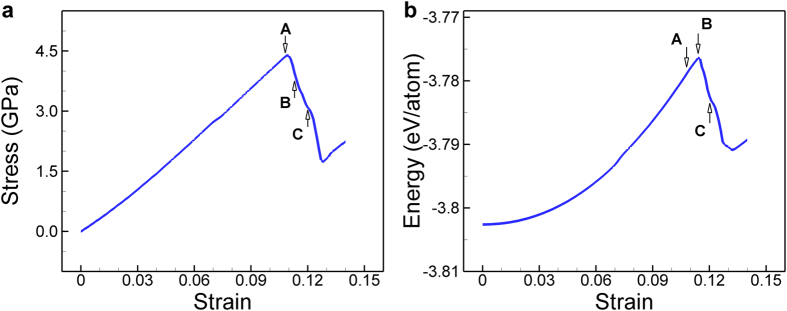
Stress and energy curves of a square Au[100]/(001) nanowire. (**a**): Stress-strain curve and (**b**): energy-strain curve. Stress starts to drop whereas energy is still increasing at point A. Cross-section of the nanowire is 3 nm × 3 nm and temperature is 0.01 K.

**Figure 2:**
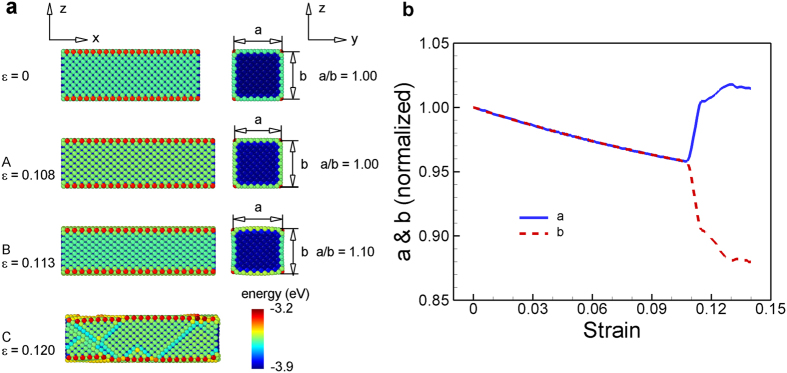
Deformation of the Au [100]/(001) nanowire under tensile loading (**a**): Snapshots of the nanowire at different strains. (**b**): Change of nanowire widths in the lateral directions. Bifurcation takes place at a strain of 0.108 and local deformation starts from a strain of 0.114. Cross-section of the nanowire is 3 nm × 3 nm and temperature is 0.01 K.

We plot the stress and energy curves to strain of a Au (001) nanowire in Fig. 1. The cross-section of the nanowire was 3 nm × 3 nm and the target temperature was 0.01 K. The stress and energy are increasing monotonously as strain increases. The stress reaches the maximum value of 4.4 GPa at a strain of 0.108 as pointed by “A” in Fig. 1a. This value can be regarded as the ideal tensile strength of the nanowire. It is remarkable that the energy is still increasing after the maximum point of stress and there is no visible change near point “A” in the energy curve, as shown in Fig. 1b. Although the stress drops significantly, there is no sudden change in the energy of nanowire. Rather, the strain energy of the nanowire reaches to the maximum value at a strain of 0.113 as pointed by “B” in Fig. 1b, and starts to decrease immediately after point “B”. In Fig. 2a, we present the configurations of the nanowires at some important points that are marked at Fig. 1. From the initial configuration (*ε* = 0) to the configuration where the stress is the maximum (*ε* = 0.108), the square nanowire is contracting in both lateral directions (the y- and z-directions) by the same amount, and thus the shape of cross-section maintains square. In many previous reports^2^,^26^,^27^,^28^, the maximum stress has been regarded as the yield stress of the nanowire, because it has been believed that the stress drops due to local failure deformations such as slip, partial slip or twinning that are closely related to the motion of dislocations. However, as shown in the third panel (*ε* = 0.113) in Fig. 2a, even though the applied strain exceeds the point at which the stress is the maximum, none of such local deformations is observed. Instead, the deformation that makes the drop of stress is a homogenous deformation. Unlike local deformation resulting from the dislocation that initiates at a point and propagates, the homogenous deformation (or the global deformation) takes place in the entire nanowire simultaneously. During this global deformation, sudden expansion in a lateral direction (here in the *y*-direction) and sudden contraction in the other lateral direction (here in the *z*-direction) occurs simultaneously. Now, the shape of cross-section is not square but a similar shape of a rectangle. At *ε* = 0.113, the ratio of *a*/*b* becomes 1.1 as shown in Fig. 2a. When we applied further strain, a local deformation was observed (*ε* = 0.120). The changes in the sizes of nanowire in the lateral directions are shown in Fig. 2b. The thicknesses in both directions were gradually reducing until the stress reached to the maximum and exhibited different changes by a relatively large amount during the stress drop.

### Failure mode of metal nanowires and bulk metals

This global deformation has been predicted in many cubic bulk materials under uniaxial loading. This phenomenon in cubic bulk materials is called as branching or bifurcation of crystal structures and has been explained by the elastic instability theory. The theory was initially developed by Born^14^, and then improved by Hill and Milstein^15^,^16^ and others^17^,^18^,^19^. For an arbitrary ideal crystal structure under an arbitrary loading mode, the necessary and sufficient condition for the stability of the structure is 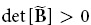, where 
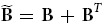
and **B** is the elastic stiffness matrix in the Voigt notation. When 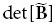 becomes zero, the crystal structure loses its stability and deforms with the corresponding eigenstate. One can obtain the corresponding eigenstate that describes the deformation of the structure at the instability point by solving an eigenvalue problem: 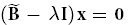. For FCC bulk crystals under uniaxial tensile loading (*σ*_1_ > 0), the general condition is simplified to *C*_22_−*C*_23_ > 0, where **C** is the elastic modulus matrix of the structure. The corresponding eigenstate to this instability condition is (*δε*_1,_*δε*_2_,*δε*_3,_*δε*_4,_*δε*_5,_*δε*_6_) = (0,1,−1,0,0,0), i.e. the deformation with a sudden expansion in a lateral direction and a sudden contraction in the other lateral direction. The magnitude of expansion is the same with the magnitude of contraction (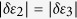). The elastic moduli of materials are varying with strain. Therefore, when *C*_22_ becomes equal to *C*_23_ at a certain strain, the FCC crystal structure loses its stability and the bifurcation occurs in the lateral directions. The bifurcation of the FCC crystal structure is actually a phase transformation from tetragonal to orthorhombic. This phenomenon was observed computationally in previous reports^29^,^30^. We confirmed that the phase transformation takes place at a critical strain of 0.088 at which the condition *C*_22_ = *C*_23_ meets for a Au (001) bulk material. Again, the stress drops at this strain but the energy does not change. The elastic instability theory predicts well the failure behavior of cubic bulk materials.

**Figure 3:**
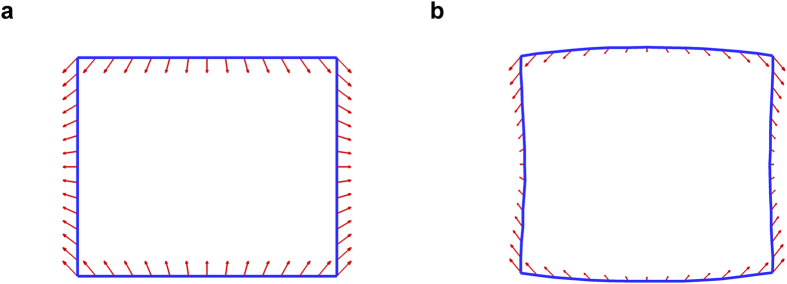
Details in the deformation during a global failure mode. Cross-sectional views of (**a**) Au (001) bulk crystal and (**b**) Au [100]/(001) nanowire at 0.01K. The cross-sectional shape of the bulk crystal becomes a right rectangle during the global deformation, but that the nanowire is not exactly rectangular because of different relaxations of atoms on edges and surfaces. Arrows denote the direction and magnitude of deformation of the atoms positioned on blue lines.

For both FCC bulk and nanowire structures under uniaxial tensile loading, they eventually undergo the bifurcation: a contraction in one lateral direction and an expansion in the other lateral direction. However, details of their deformations at the bifurcation are different from each other, because there are substantial effects of free surfaces and edges for nanowires. Under uniaxial tensile loading, only one normal component of stress is nonzero (positive) and the others are zero. Under this loading condition, all the atoms in a bulk material have the same stress-state and the total stress is also the same: only one nonzero normal component of the stress. However, for nanowires, whereas the total stress has only one nonzero component, the stress state of each atom is different depending on its position in the nanowires. For FCC nanowires, there is a large relaxation of free surfaces and edges, and thus each atom is redistributed according to the relaxation. As a result, all atoms of the nanowires have three or more nonzero components of stress, and further the magnitudes of nonzero component are different. Stress is usually more tensile at the outmost atomic layers and less tensile at the interior part. Therefore, atomic displacements of the nanowires before and after the bifurcation are dependent on the positions of atoms, and thus they are different from those of bulk materials. Consequently, after the failure, the cross-section of the nanowire is not exactly rectangular like the case of bulk, as shown in Fig. 3b. The deformation at the edges is approximately three times larger than those at the middle part of the free surfaces.

These different displacements or stresses of atoms in nanowires make it difficult to predict the instability moment by adopting the elastic instability theory, because atomic elastic moduli of the nanowires also become functions of atomic position. The elastic moduli at surfaces or edges are different from those in the interior part. Therefore, we need to develop an equivalent elastic modulus that can predict the instability phenomenon of the nanowires. Among possible choices, we replaced the condition, *C*_22-_*C*_23_ > 0, by the condition, 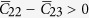, where 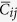 is an equivalent elastic modulus of the nanowire given by 
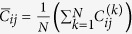
 where *N* is the total number of atoms and 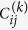
is the elastic modulus of atom *k*. Therefore 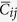 is just the average value of the elastic modulus of all atoms. We found that the term 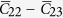 was approximately 34 GPa at the initial configuration, and it decreased almost linearly with the increase of strain. At the bifurcation point, the term became approximately 0.97 GPa that was relatively small in comparison with the initial value. When we introduced 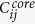, which was the elastic modulus of the atoms at the center of the nanowire, for the equivalent elastic modulus, 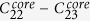 reduced from 37 GPa at the initial configuration and reached to 1.06 GPa at the configuration in which a bifurcation took place. Although there is more possibility in the choices for the equivalent elastic modulus to improve the prediction of the failure moment of nanowires, the elastic instability theory still predicts well the bifurcation of metal nanowires.

The global deformation (bifurcation) takes place not only in Au [100]/(001) nanowires but also in other FCC metal [100]/(001) nanowires. We performed the same MD simulations to study the failure mode of other nanowires, namely Al, Ni, Cu, Pd, Ag, and Pt. All of the metal nanowires considered showed the same global deformation as their failure modes. Furthermore, in order to confirm the generality of the global deformation of FCC (001) nanowires as the failure mode, we employed three different interatomic potentials such as Cai and Ye^21^, Foiles *et al.*^20^, and Liu *et al.*^22^. Regardless of types of interatomic potentials employed, the global deformation always took place. The generality of the global deformation as the failure mode was also supported by the fact that all of Au [100]/(001) nanowires with various thicknesses ranged from 2.2 nm × 2.2 nm to 8.4 nm × 8.4 nm exhibited the same global deformation.

The global deformation, which is the simultaneous occurring of the sudden expansion in a lateral direction and sudden contraction in the other lateral direction, is the unique property of FCC (001) nanowires under tension. This is because the deformation mode at onset of instability (*δε*_1,_*δε*_2_,*δε*_3,_*δε*_4,_*δε*_5,_*δε*_6_) = (0,1,−1,0,0,0) can only be found in cubic materials under uniaxial stress or hydrostatic loading condition. In BCC (001) structures, this deformation mode takes place under uniaxial compressive stress condition. It is noteworthy that when we consider FCC structures with other crystallographic directions such as (111) or (110), the deformation mode becomes a function of elastic moduli unlike (001) structures^31^. In addition, this deformation mode is not clearly shown when the structures become nanowires owing to the additional surface effects.

So far, we considered the simulations with the strain rate of 10^8^ s^−1^. It has been known that mechanical properties of nanowires are dependent on the strain rate^32^,^33^,^34^. For example, the yield strength of nanowires decrease with decrease of the strain rate^2^. Dynamic wave effect at high strain rate may prevent the nucleation of defects in nanowires^35^ and therefore the nucleation stress of dislocations becomes smaller as strain rate is lower. In order to confirm our finding, we conducted MD simulations for [100]/(001) nanowires of six metals, namely Cu, Ag, Au, Ni, Pd, and Pt with the EAM potential by Foiles, with the strain rate range of 10^6^-10^8^ s^−1^ at low temperature. In addition, quasi-MD simulations were also employed at low temperature. Through the simulations we found the global deformation as the failure mode for all cases. Therefore, we can conclude that it is possible to observe the global deformation as a deformation mode of metal [100]/(001) nanowires regardless of strain rate.

### Global deformation versus local deformation

**Figure 4:**
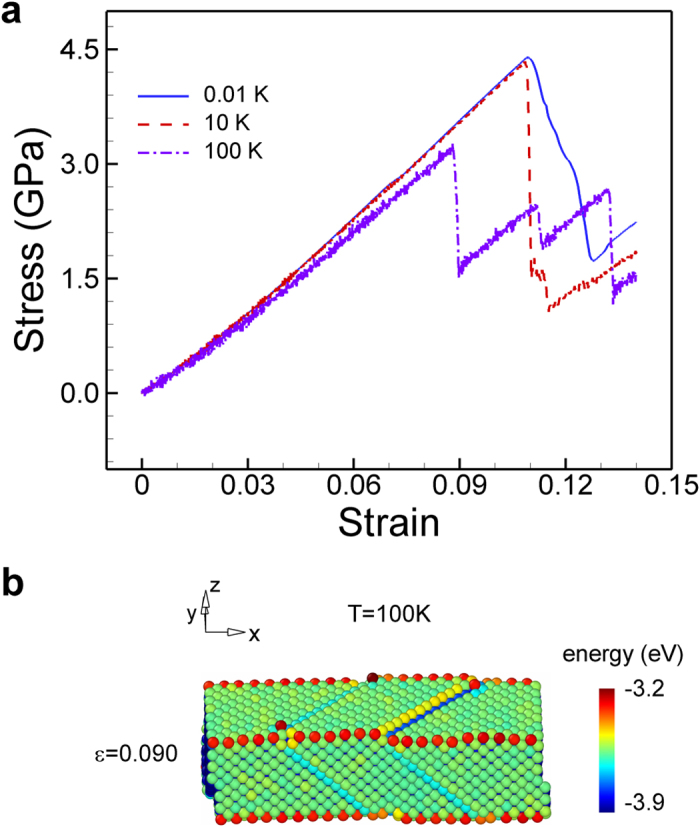
Effects of temperature on the mechanical response of a Au[100]/(001) nanowire. (**a**) Stress-strain curve and (**b**) snapshot immediately after stress drop at 100 K. At low temperature a global deformation takes place whereas a local deformation occurs at high temperature. Cross-section of the nanowire is 3 nm × 3 nm.

So far, we have shown that the global deformation induced by the elastic instability is the failure mode of FCC (001) nanowires. Then, a couple of questions arise: why do the simulations show global deformation as the failure mode instead of the local deformation like many other reports? Do the observed results violate the previous studies? In order to answer the important questions, we performed the tension tests of a Au [100]/(001) nanowire at different temperatures: 0.01 K, 10 K, and 100 K. Figure 4a presents the stress-strain curves of the nanowire under tensile loading at different temperatures. At lower temperatures (0.01 K and 10 K) the global deformation took place, while the nanowire failed with a local deformation at smaller strain (*ε* = 0.090) at higher temperature (100 K), as shown in Fig. 4b. The local deformation at 100 K was the combination of full and partial slips, which was in agreement with the reported result by Diao *et al.*^3^. In this case, the maximum stress can be regarded as the yield stress of the nanowire, because the nanowire fails with a yield event. However, at low temperature, the nanowire failed with a global deformation, and now the maximum stress is the ideal tensile strength rather than the yield stress. Based on the observation, we may conclude that the nanowire fails homogenously at low temperature which had never reported in literature before, while it fails partially at higher temperature which is commonly observed in previous studies.

Local deformations are mainly the results of the nucleation and propagation of dislocations. The required stress for the nucleation of dislocations is critical for the occurrence of the local deformation, because the required stress for the propagation of dislocation is usually very low in metals. It has been known that the nucleation stress for dislocation is dependent on temperature: increase of temperature lowers the nucleation stress^13^. Therefore, at low temperatures, global deformation takes place sooner than local deformation because the nucleation stress is much higher than the stress at the instant of elastic instability. In contrast, at high temperature, a local deformation occurs first, because the nucleation stress is lower than the stress at the bifurcation. It is worth noting that, even at high temperatures (>300 K), we always observe the global deformation as the failure mode of (001) bulk materials rather than the local deformation, because the nucleation stress of dislocations in the bulk materials is much larger than the stress for global deformation. This means that, at low temperature (~10 K), the nucleation stress of dislocations at the edges of nanowires lies in a similar range of the stress for global deformation. Therefore, one can expect that the global deformation would be the failure mode even at higher temperatures (> 10 K), when edge is removed.

**Figure 5:**
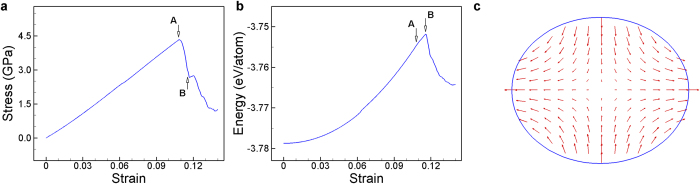
Tensile loading test of a circular Au (001) nanowire. (**a**): Stress-strain curve, (**b**): energy-strain curve, and (**c**) deformation at point B during a global failure mode. Stress starts to drop, whereas energy is still increasing at point A. Diameter of the nanowire is 3 nm and temperature is 0.01 K. Arrows denote the direction and magnitude of deformation of the atoms.

We have discussed that the nanowires with a square cross-section exhibit the global failure mode at low temperature. As temperature increase, local failure mode becomes dominant. The temperature at which the failure mode changes is approximately 12 K for a square Au nanowire (with the EAM potential by Cai and Ye) with a size of 3 nm × 3 nm. This transient temperature depends on the cross-sectional shape of nanowires. In order to investigate the effects of the shape of nanowires on the failure mode or the transient temperature, we performed the same MD simulations for a circular Au nanowire with a diameter of 3 nm. We changed the temperature of the circular nanowire from 0.01 K to 200 K. First, at 0.01 K, the circular nanowire failed with a global deformation at the critical strain of 0.108, which was the same with the critical strain of the square nanowire as shown in Fig. 5. It is noteworthy that, in the previous study by Cao and Ma^4^, the yield stress of a circular nanowire was found to be 50% larger than that of a square nanowire with a similar size. However, the yield stresses, under which a local deformation occurs, were compared in their study. And, the nucleation stress of the dislocations that are responsible for the local deformation is different depending on the nucleation sites: edges for the square nanowire and surfaces for the circular one. Therefore, the yield stresses of the two nanowires should be different. In contrast to their study, here, both nanowires failed with a global deformation when their equivalent elastic moduli satisfied the instability condition, 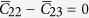. We believe that the elastic moduli of the circular nanowire and the square one are almost the same at certain temperature and loading. Hence, if both nanowires fail with a global deformation, they should fail at almost the same critical strain as shown in Fig. 5.

### Transient temperatures

Next, we found that the transient temperature of the circular nanowire is approximately 100 K, which is much higher than that of the square nanowire, 12 K. As discussed earlier, the nucleation stress of dislocations on the edges of a nanowire is lower than that on the surfaces of the nanowire. This is also supported by the study of Cao and Ma^4^ on the effects of the shapes of nanowires on the yield strength of the nanowires. Using a strain shear invariant, they found that the edges of nanowires are the easiest sites for the nucleation of dislocations. Further, Zhu *et al.*^13^ showed that a square nanowire has 6 times smaller barrier than a flat surface of the same material for the nucleation of dislocations. All the previous reports support the fact that, at a certain temperature, the nucleation stress of dislocations in circular nanowires is larger than that in square nanowires with a similar size, because of the absence of edges in circular ones. Thus, circular nanowires undergo more elastic deformation than square nanowires until dislocations nucleate. In other words, there is much more possibility for circular nanowires that they meet the elastic instability condition before the stress in them reaches the nucleation stress. On the other hand, because the nucleation stress is small at edges, dislocations can initiate from the edges of square nanowires before the nanowires reaches to their elastic instability condition.

**Figure 6:**
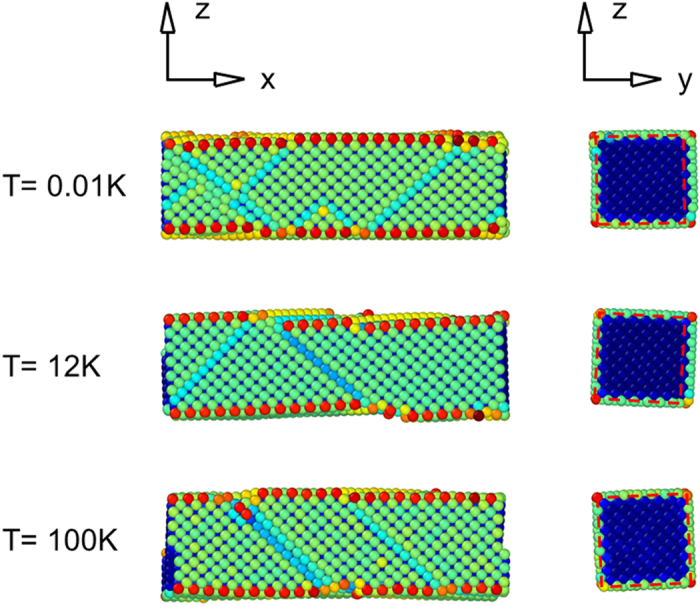
Snapshots of a Au [100]/(001) nanowire at a strain of 0.120. The cross-sectional views are shown on right panels. Red dashed lines denote the same square. At the transient temperature (~12 K), both of the local and global deformations take place simultaneously. Cross-section of the nanowire is 3 nm × 3 nm.

Even though nanowires fail with a global deformation at low temperature, a local deformation takes place at last following the global deformation when more tensile loading is applied to the nanowires. Figure 6 shows configurations of the Au nanowire with different temperatures at a strain of 0.120. In all the configurations, local deformations were observed. At 0.01 K, the nanowire underwent a global deformation at a strain of 0.108, and a local deformation followed from a strain of 0.114 with a significant drop in the strain energy of the nanowire (Fig. 1a). In contrast, the nanowire failed with a local deformation at a strain of 0.090 and no global deformation was observed at 100 K. As a result, the cross-sectional shape of the nanowire was similar to a rectangle at 0.01 K, whereas it was similar to a square at 100 K, as shown in right panels in Fig. 6. It is noteworthy that the cross-sectional views in Fig. 6 were a little bit rotated because local deformations (partial cross-slips) accompanied a small degree of rotations.

Interestingly, at near the transient temperature which was approximately 12 K for this nanowire, we observed that the global and local deformations occurred simultaneously. At this temperature, the required stresses for the nucleation of dislocation and for the elastic instability are very similar to each other, and thus the nanowire undergoes both deformation modes simultaneously during the failure. At low temperatures (<12 K), global deformation takes place first and thus mechanical properties of the nanowire changes significantly during deformation. For examples, two extended surfaces by bifurcation of the nanowire are mechanically weaker than the other two contracted surfaces. As a result, later local deformation is determined by new mechanical properties. At near the transient temperature (~12 K), we observed that a dislocation initiated from one edge of the nanowire and propagated while the expansion and contraction of the nanowire along the lateral directions were taking place.

**Figure 7:**
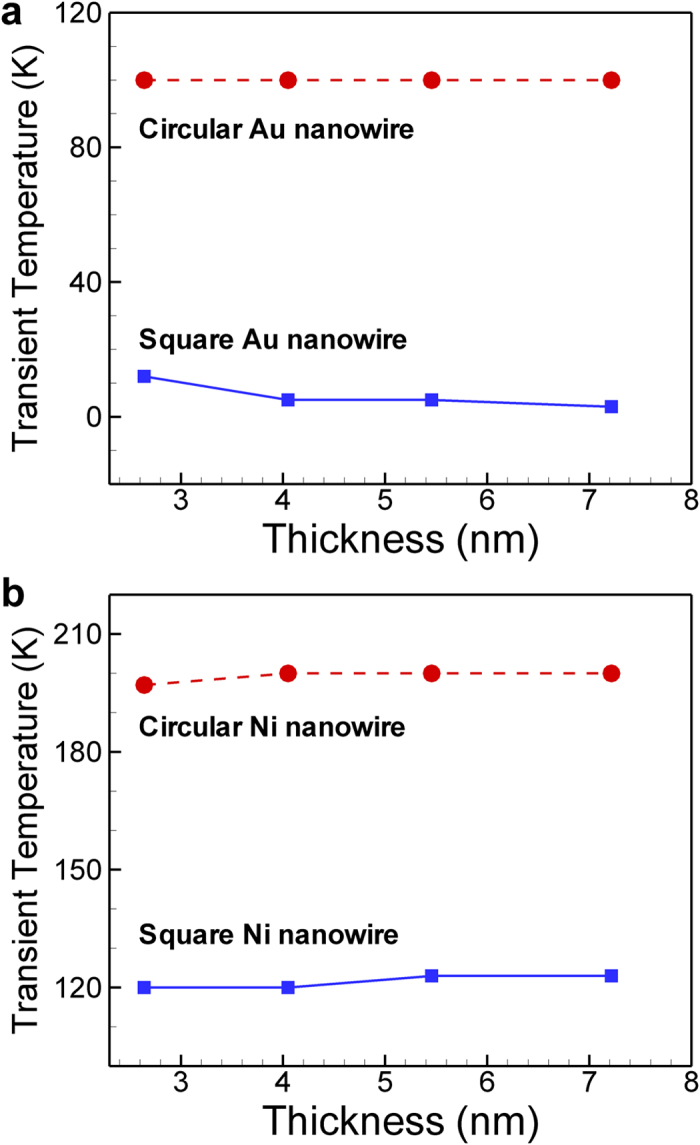
Comparison of transient temperatures for (001) nanowires (**a**): Au nanowires described by the Cai and Ye’s potential and (**b**): Ni nanowires described by Foiles *et al.*’s potential. Circular nanowires exhibit much higher transient temperature than square nanowires owing to the absence of edges. The transient temperatures of Ni nanowires are approximately 100 K higher than those of Au nanowires.

For all the seven metal (001) nanowires and the three different interatomic potentials we employed, there always exist the transient temperatures under which the global deformation is the failure mode for the nanowires and near which the global and local deformations occur simultaneously. However the transient temperatures strongly depends on the FCC metals and the interatomic potentials. For examples, we compared the transient temperatures of Au and Ni nanowires with different lateral sizes and cross-sectional shapes in Fig. 7. Here, the EAM potential developed by Cai and Ye was employed for the Au nanowires while different EAM potential developed by Foiles *et al.* was used for the Ni nanowires. The transient temperatures of circular nanowires were much higher than those of square nanowires for both metals, because of the absence of edges in the circular nanowires as discussed previously. And, although the transient temperatures slightly differed as thickness increases, they did not show a clear dependency on the thickness. On the other hand, it is remarkable that the transient temperatures of the square Au nanowires were extremely low (<15 K) whereas those of the square Ni nanowires were much higher (~120 K). The transient temperatures of circular Ni nanowires were approximately 200 K which was approximately 100 K higher than those of circular Au nanowires. The transient temperatures we obtained through MD simulations are much lower than room temperature and thus it is hard to observe the global deformation as a failure mode because it takes place at temperatures lower than the transient temperature. However, we believe that it is still meaningful that there is another failure mode for FCC (001) nanowires at extremely low temperature.

## Discussion

We performed MD simulations to study the failure behavior of FCC (001) metal nanowires under uniaxial loading. We found that global deformation is a mechanical failure mode of nanowires under tension besides the commonly believed local deformation mode. At the failure of nanowires with the global deformation, a large expansion along a lateral direction and a large contraction along the other lateral direction take place simultaneously in the nanowires. Such phenomenon is driven by the elastic instability of materials. We also found that there is a competition between the global deformation mode induced by the elastic instability and the local deformation mode induced by the nucleation and propagation of dislocations. At low temperatures, the global deformation mode is dominant because the thermal activation is not enough to nucleate dislocations. At high temperatures, nanowires meet the condition for the nucleation of dislocations before they meet the elastic instability conditions and thus local deformation becomes dominant. At near the transient temperatures, nanowires fail by the simultaneous occurrence of the global and local deformations.

Although we found that there are large differences in the transient temperatures of FCC (001) nanowires depending on the metals, all transient temperatures we obtained in MD simulations were much lower than room temperature. Therefore, we expect that FCC (001) nanowires under tensile loading will always fail by local deformations in real experiments at room temperature. However, it would be possible that one verify the global deformation as a failure mode of FCC (001) nanowires by experiments at extremely low-temperature.

## Additional Information

**How to cite this article**: Ho, D. T. *et al.* Mechanical Failure Mode of Metal Nanowires: Global Deformation versus Local Deformation. *Sci. Rep.* **5**, 11050; doi: 10.1038/srep11050 (2015).
